# Rapid 16S rRNA Next-Generation Sequencing of Polymicrobial Clinical Samples for Diagnosis of Complex Bacterial Infections

**DOI:** 10.1371/journal.pone.0065226

**Published:** 2013-05-29

**Authors:** Stephen J. Salipante, Dhruba J. Sengupta, Christopher Rosenthal, Gina Costa, Jessica Spangler, Elizabeth H. Sims, Michael A. Jacobs, Samuel I. Miller, Daniel R. Hoogestraat, Brad T. Cookson, Connor McCoy, Frederick A. Matsen, Jay Shendure, Clarence C. Lee, Timothy T. Harkins, Noah G. Hoffman

**Affiliations:** 1 Department of Laboratory Medicine, University of Washington, Seattle, Washington, United States of America; 2 Department of Genome Sciences, University of Washington, Seattle, Washington, United States of America; 3 Department of Microbiology, University of Washington, Seattle, Washington, United States of America; 4 Life Technologies, Beverly, Massachusetts, United States of America; 5 Public Health Science Division, Fred Hutchinson Cancer Research Center, Seattle, Washington, United States of America; University of Aberdeen, United Kingdom

## Abstract

Classifying individual bacterial species comprising complex, polymicrobial patient specimens remains a challenge for culture-based and molecular microbiology techniques in common clinical use. We therefore adapted practices from metagenomics research to rapidly catalog the bacterial composition of clinical specimens directly from patients, without need for prior culture. We have combined a semiconductor deep sequencing protocol that produces reads spanning 16S ribosomal RNA gene variable regions 1 and 2 (∼360 bp) with a de-noising pipeline that significantly improves the fraction of error-free sequences. The resulting sequences can be used to perform accurate genus- or species-level taxonomic assignment. We explore the microbial composition of challenging, heterogeneous clinical specimens by deep sequencing, culture-based strain typing, and Sanger sequencing of bulk PCR product. We report that deep sequencing can catalog bacterial species in mixed specimens from which usable data cannot be obtained by conventional clinical methods. Deep sequencing a collection of sputum samples from cystic fibrosis (CF) patients reveals well-described CF pathogens in specimens where they were not detected by standard clinical culture methods, especially for low-prevalence or fastidious bacteria. We also found that sputa submitted for CF diagnostic workup can be divided into a limited number of groups based on the phylogenetic composition of the airway microbiota, suggesting that metagenomic profiling may prove useful as a clinical diagnostic strategy in the future. The described method is sufficiently rapid (theoretically compatible with same-day turnaround times) and inexpensive for routine clinical use.

## Introduction

In nature, microbes exist in complex communities shared with other species rather than as pure cultures dominating an ecological niche. The microbiota in healthy humans [1], [2] and in various human disease states, ranging from chronic infections [3] to autoimmune disorders and metabolic disease [4], are no exception, frequently cohabitating organ systems or acting in concert as polymicrobial biofilms. Nevertheless, the ability of existing methods in clinical microbiology to rapidly enumerate and thoroughly classify the diversity of organisms present in such patient specimens is lacking.

Traditional microbiological classification is rooted in organisms' morphology and biochemical properties and first requires that species are isolated by growth *in vitro*. Only a small fraction of all bacteria can be successfully cultured, while clinically significant organisms may be slow-growing, fastidious, inert, or unviable [5]. Individual strains may out-compete others when co-cultured, and overwhelming numbers of species may be present, prohibiting a comprehensive workup. 16S ribosomal RNA (rRNA) gene sequencing is a popular alternative to traditional methods and provides several advantages [6], [7]. DNA sequencing can provide more definitive taxonomic classification than culture-based approaches for many organisms [6], [7], while proving less time consuming and labor intensive [6], [8]. However, 16S rRNA gene sequencing using bulk PCR products cannot be applied to polymicrobial specimens: the presence of multiple templates results in superimposed Sanger reads that are generally uninterpretable [8], [9].

As first realized through metagenomics research [10], next-generation sequencing technologies [11] can circumvent these inherent limitations. Aside from benefits in per-base sequencing costs and throughput, deep sequencing methods provide individual sequence data for millions of DNA molecules, allowing each to be classified independently. Regardless, next-generation 16S rRNA gene sequencing methods have not been utilized in clinical microbiology practice due to barriers in sequencing costs and procedural challenges including the time and effort required to prepare and sequence libraries and the complexity of the analysis; these objectives must be completed within a timeframe that can meaningfully inform patient care.

Here, we develop a rapid and inexpensive culture-free next-generation sequencing assay able to accurately catalog bacterial species directly from highly complex patient specimens by 16S rRNA gene deep sequencing. As a proof of principle, we explore the utility of this assay in comparison to existing clinical microbiology techniques across a collection of challenging clinical samples and cystic fibrosis sputum samples.

## Materials and Methods

### Ethics Statement

Although human-derived samples were used in this study, this work is not considered human subjects research, and is not considered to involve human participants per University of Washington Human Subjects Division because the material constituted non-identifiable, leftover clinical specimens that were not collected specifically for this study (UW IRB Doc #295). As this work is not human subjects research and does not involve human participants, this work is exempt from needing ethical approval and written informed consent:

“Use of Non-Identifiable Specimens/Data . . .requires neither determination of exempt status nor IRB review” (UW IRB Doc #295).

Per UW IRB Document #359:

“2.4.2 The UW IRB interprets this definition to mean that a human specimen falls within the definition of health care information only when:

- It can be tied to an individual, and
- It was obtained in the course of diagnosing, treating, or otherwise providing health care in the state of Washington.

2.4.3 This means that a human specimen is not, in and of itself, considered to be a human subject by state law, unless it can be readily connected to an individual.”

Only “If your research activity involves human subjects, it is necessary to complete the appropriate HSD form for submission, review, and approval prior to commencement of the research activity.”

Use of leftover, non-identifiable patient samples is ‘Not Human Subjects Research’ as defined by the UW IRB, and does not require UW IRB approval (UW IRB Doc #295).

We completed and filed with the UW IRB a self-determination form for approval of Use of Non-Identifiable Specimens/Data, which was approved by the Department Chair of Laboratory Medicine, and which states that “the project requires neither determination of exempt status nor IRB review” (UW IRB Doc #295).

With specific respect to written informed consent: Per UW IRB review (UW IRB Doc #295 and #359, as cited above), this work did not involve human participants, and thus, no waiver for the need of written informed consent was required. The University of Washington IRB board has deemed this research “Not Human Subjects Research”, therefore does not involve human participants (UW IRB Doc #295).

### Samples and DNA purification

Microbiological culture, isolation and identification of species were performed by the University of Washington Clinical Microbiology Laboratory, according to standard clinical procedures. Briefly, samples submitted for “Lower Respiratory Culture for Cystic Fibrosis” (referred to hereafter as “CF sputum samples”) were mixed 1∶1 with 0.0648 M dithiotreitol (Sigma) and incubated for 5 minutes at room temperature, followed by vigorous vortexing for 1 minute. 50 µl aliquots of mucolysed sputa each were plated on sheep blood agar, MacConkey, chocolate, manitol salt, and cepacia agar culture plates. The remaining specimen was stored at −20°C until DNA extraction was performed.

DNA was extracted from isolated colonies or from the remaining volume of mucolysed sputa using a NucliSENS Easymag automated DNA extractor (BioMerieux). For CF sputa samples we included one extraction control per batch of 24 samples processed simultaneously. Abscess and lymph node biopsy material were purified with High Pure PCR Template Preparation Kit (Roche). Extracted DNAs were quantified by Qubit dsDNA HS kit (Life Technologies). For mixing studies, the relative contribution of 16S rRNA alleles from each organism was estimated from quantified input DNA, average genome size of sequenced reference strains, and average 16S rRNA locus copy number for the species. A mixture of 16S rRNA template from the following organisms was used: *P. aeruginosa* , 80%; *B. cepacia* , 14.11%, *S. pyogenes*, 5.65%; *M. tuberculosis*, 0.24%.

Bacterial genomic DNA isolated from isolates of clinical specimens was sequenced by the University of Washington Molecular Microbiology Laboratory using the Sanger method to establish 16S rRNA gene reference sequences or to attempt molecular diagnosis, where applicable.

Three clinical specimens were excluded from the final analysis. CF13 and CF46 each generated too few de-noised bacterial sequence reads for meaningful analysis (2682 reads and 411 reads, respectively), likely secondary to poor balancing of the libraries. CF90 was also excluded, as we could not adequately confirm the identity of this specific specimen.

### Target Selection

The 16S rRNA gene contains nine variable regions (designated V1 to V9) [12]. Here we chose the V1–V2 region, which has previously proven useful in research-oriented metagenomic surveys [13]–[15] and is used clinically for conventional sequencing-based classification assays, because it can provide species-level classification of clinically-relevant bacteria, permits selective exclusion of contaminating eukaryotic sequences (which share homology with some conserved regions of prokaryotic 16S rRNA genes) from PCR amplification [16], and is a relatively small fragment (∼360 bp) that permits PCR amplification from partially degraded specimens.

### Sequencing library generation

The sequences of PCR primers used for library preparation (Integrated DNA Technologies, PAGE purified) are supplied in **Table S1**. All PCR setup was performed in a laminar flow PCR workstation, and materials were UV irradiated prior to PCR setup. PCR to amplify 16S rRNA was carried out in two stages. Prior to the second stage, unincorporated primer was removed by DNA purification and additional cycles of PCR were performed using primers specific to the sequencing adaptor, amplifying only the molecules generated during initial PCR cycles. We found that this two-stage PCR strategy greatly reduces the amount of amplification from non-template controls.

For the first round of PCR, a primer directed against the V1 flanking region (Primer P1) was used in conjunction with forward primers incorporating (from 5′ to 3′) Ion Torrent sequencing adaptor P1, a sample-specific “DNA molecular tag”, a 14-base semirandom sequence (intended to uniquely identify original template molecules [17], but not utilized in these studies), and lastly a universal bacterial primer directed against the 16S rRNA V2 flanking region (Primer *N*_Barcode_357mI). To minimize amplification of contaminating bacterial DNA present in PCR reagents [18], [19], 1∶10 diluted AmpliTaq DNA polymerase (Applied Biosystems) was used during two initial cycles of PCR amplification [19]. PCR was conducted using a 0.9 µM concentration of each primer, and 1–10 ng DNA template according to the following cycling conditions: one cycle of 95° for 10 minutes, two cycles of 95° for 30 seconds, 55° for 30 seconds, 72° for 1 minute 15 seconds, then one cycle of 72° for 10 mintues. Amplification products were purified using 0.7 volumes of Agencourt AMPure beads (Beckman Coulter), without removing the beads after elution. The second round of PCR was carried out using the recommended concentration of AmpliTaq with a 0.44 µM concentration of each primer. Primers for this step were composed of the Ion Torrent paired-end sequencing adaptor P1 joined to the V1-targeted primer (P1_PE_Adaptor) and a 5′ fragment of Ion Torrent sequencing adaptor A (Universal_357mI_Primer). The entire volume of purified amplicon from the first PCR reaction was amplified according to the following cycling conditions: once cycle of 95° for 10 minutes, 35 cycles of 95° for 30 seconds, 68° for 30 seconds, 72° for 1 minute 15 seconds, then one cycle of 72° for 10 mintues. Final PCR products were purified with 0.7 volumes Agencourt AMPure beads, eluted in low TE, and quantified by Qubit dsDNA HS kit. Equal quantities of PCR product from each sample were pooled for sequencing (Table S2), and the final concentration of each library was determined using a Bioanalyzer High Sensitivity DNA Kit (Agilent).

### Semiconductor Sequencing

Sequencing was performed by Life Technologies (Beverly, MA). The sequencing protocol was under development, particularly during the timeline of this project, and the details of the procedure had not been fully optimized for commercial release of 400 bp sequencing kits. For emulsion PCR, the protocols for the Ion PGM™ 200 Xpress™ Template Kit (Life Technologies) were modified to accommodate clonal amplification of the sequencing templates on to Ion Sphere Particles (ISPs, Life Technologies). The amount of ISPs and library molecules added to the emulsion was increased by 55%. A new polymerase and changes in salt conditions were also required for full extension of the longer template reads. PCR thermocycling conditions were modified as follows: one cycle of 95° for 6 minutes, 15 cycles of 95° for 30 seconds, 68° for 4 minutes, 30 cycles of 95° for 30 seconds, 68° for 6 minutes, then 10 cycles of 95° for 30 seconds, 68° for 20 minutes. Enrichment and quantification of template beads was performed according to manufacturer protocols.

400 bp semiconductor sequencing also required optimization in sequencing workflow and chemistries. A proprietary sequencing enzyme has been developed to increase both accuracy and read lengths, with concurrent optimization of flow order and nucleotide flow rates. Sequencing was performed on an Ion Torrent PGM (Life Technologies) using 800 flows (200 cycles), as opposed to the standard 520 flows. All sequencing was performed using 318 chips, with an approximate runtime of 7 hours per chip. Primary base calling was performed using Torrent Suite v3.0 software (Life Technologies), and sequences were exported in FastQ format. FastQ files were used for all subsequent analyses. Raw sequence reads for this project are available from the Sequence Read Archive (http://www.ncbi.nlm.nih.gov/sra), under study accession number SRP019805.

Sequencing reagents and protocols have subsequently been optimized and are available as Ion Torrent 400 bp sequencing kit (Life Technologies).

### Data processing and de-noising

We required that reads exceed 330 base pairs in length and contain one or fewer mismatches against a barcode sequence to pass initial filtering. Primer sites were identified in each read using the Smith-Waterman alignment algorithm (ssearch36) [20] with the requirements that sequence regions corresponding to forward and reverse PCR primer sites appeared in specified flow position windows and primer alignments exceed a threshold Z-score of 100, defined based on visual inspection of alignments and corresponding distributions of Z-scores. Reads not meeting these criteria were discarded, and remaining reads were trimmed to exclude primer sites.

De-noising of trimmed reads was accomplished by (1) performing a modified [21] form of run-length encoding [22], in which each homoploymer is replaced by a single nucleotide and the homopolymer length is recorded; (2) clustering the encoded reads at 98.5% identity using *USEARCH* v6 [23]; and (3) creating multiple alignments of encoded reads comprising clusters of three reads or greater using *MUSCLE* v3.5 [24]. To minimize computational time, clusters of greater than 100 reads were randomly grouped into smaller sets of 100 to no more than 150 sequences and each was aligned separately. (4) A consensus was generated from each multiple alignment by expanding the most frequent character at each position by the most frequent run-length for that character. (5) Identical consensus sequences were aggregated and the total number of reads representing each was recorded.

Parameters for de-noising were chosen empirically by calculating error rates as described below using sequences generated from control specimens containing a mixture of reference organisms with known 16S rRNA gene sequences (**Figure S1**). Parameters for de-noising were selected to maximize both the number of recovered reads and the pairwise identity of those reads compared to the appropriate reference sequence. We found that a clustering threshold of 98.5% or greater pairwise similarity combined with exclusion of clusters composed of fewer than 20 reads resulted in the most favorable combination of error rate and read recovery. For evaluation of non-template and extraction controls, clusters composed of 10 reads or greater were considered in order to further increase sensitivity.

De-noised reads from this project are available in **File S1**.

### Error rate calculations

Tabulation of errors was performed by calculating pairwise alignments of either individual reads or de-noised cluster consensus reads against a reference sequences obtained from Sanger sequencing of control specimens, and counting errors in the former relative to the latter. To minimize the effect of alignment artifacts arising from homopolymer miscounting errors, we also used run length encoding to improve the quality of pairwise alignments: we run-length encoded both reference sequences and reads or consensus sequences as described above, performed pairwise alignment of encoded sequences using the Smith-Waterman algorithm (ssearch36) [20] with a gap opening penalty of 3 and a gap extension penalty of 8, then run-length decoded both sequences in the context of each pairwise alignment. Errors were categorized as follows: single nucleotide substitution, homopolymer indel (homoindel), indel in nonredundant sequence, and compound error (event involving two or more categories). To minimize errors attributable to low-levels of sequences originating from contaminating DNA in PCR reagents, we excluded raw reads having a Z-score<580 in a pairwise alignment with a reference sequence, a cutoff which we found to exclude reads that were dissimilar to reference sequences but similar to exogenous sequences based on BLAST searches against a database of 16S rRNA gene sequences (described below).

### BLAST database construction

Candidate full-length 16S rRNA gene sequences (“RDP-full-length”) were downloaded from the Ribosomal Database Project (RDP, Release 10, Update 30) [25] by selecting the options “isolates,” “good quality,” and “>1200 bp.” We created two additionally curated databases derived from these candidate sequences. The first (“RDP-named”), was generated by first removing records with non-canonical taxonomic names (for example, names indicating direct submissions of unclassified organisms), then by clustering sequences by species and rejecting records with a pairwise identity to the cluster medoid of less than 98.5%. Filtering and annotation was performed using *DeeNuRP* (https://github.com/fhcrc/deenurp), and *taxtastic* (https://github.com/fhcrc/taxtastic). The second reference database was a collection of reference sequences for the purpose of classifying CF pathogens in comparison to culture (“CF pathogens”). To further minimize mis-annotation, we compared sequences in RDP-named to a collection of sequences spanning 16S rRNA V1–V2 or V1–V3, generated from clinical isolates identified in the Molecular Microbiology Laboratory at the University of Washington Medical Center. We used all sequences from clinical isolates representing genera containing any species designated as a CF pathogen. The CF pathogens database was then constructed by retaining any full-length sequence in RDP-named with both ≥99.5% identity (with at least 99% coverage) when aligned to a clinical sequence using BLAST, and with the same species-level taxonomic label.

### Phylogenetic reference set creation

The *pplacer* suite of tools (v1.1.alpha13r2-249-g71f99d8) performs phylogenetic-based classification and population analysis by adding query sequences to a phylogenetic tree comprised of reference sequences [26]. Reference sequences are most conveniently provided in a “reference package” containing a multiple alignment and corresponding phylogenetic tree, along with taxonomic and other annotation [27]. We created two reference packages by recruiting 16S rRNA reference sequences based on similarity to denoised reads from CF specimens, and then selecting representatives of each species using “deenurp search-sequences” and “deenurp select-references” in *DeeNuRP*. Reference sequence selection for species of interest was performed by minimizing the average distance to the closest leaf (ADCL) of reads placed on a phylogenetic tree of candidate reference sequences as implemented in “guppy adcl” [27]. The first reference package (CF-named, **File S2**) was assembled from sequences in RDP-named and was used for taxonomic assignment; the second (CF-unnamed, **File S3**) was assembled by comparing denoised reads to the RDP-full-length database. Multiple alignments of reference sequences were created using *cmalign* [28], and phylogenetic tress were built with *FastTree* [29]. Reference packages were assembled using *taxtastic*.

### 16S rRNA Classification

We classified sequences using a combination of BLAST searches against curated databases of 16S rRNA sequences (RDP or custom BLAST databases, as described above) and phylogenetic-based classification using *pplacer*.

We performed high-confidence species-level classification of denoised sequences on the basis of BLAST searches [30] against either the RDP-named (for brain abscesses and lymph node biopsy), or the CF-pathogens databases (for CF sputum specimens). We took a conservative approach to assigning taxonomic names to denoised reads: we assigned each consensus sequence the taxonomic name or names of any reference sequences aligning with at least 99% pairwise identity and 95% sequence coverage. Compound names (for example “*Streptococcus mitis/oralis*”) were constructed when reference sequences representing more then one species met these criteria. Consensus sequences with no qualifying matches were designated “no match”.

To perform a more comprehensive taxonomic assignment of the CF sputum specimens, we used *pplacer* to perform phylogenetic placement of denoised reads onto the CF-named reference set described above. Multiple alignments of reads to reference alignments were created with *cmalign*. After placement, “guppy classify” was used to perform taxonomic assignment using default parameters. To remain consistent with conventions used in the clinical molecular microbiology lab for classifying closely related species, we modified the *pplacer* classification results as follows: any genus- or species-level names within family *Enterobacteriaceae* were renamed to “*Enterobacteriaceae*”; *Pseudomonas hibiscicola* was renamed to *Stenotrophomonas maltophilia*; any combination of *Streptococcus mitis*, *S. oralis*, *S. pneumonia*, or *S. pseudopneumoniae* was renamed to *S. mitis/oralis/(pseudo)pneumoniae*; any species belonging to the *B. cepacia* complex was renamed “*Burkholderia cepacia* complex”; and members of any combination of *Achromobacter denitrificans*, *A. insolitus*, or *A. xylosoxidans* was renamed to *A. denitrificans/insolitus/xylosoxidans*. In addition, reads classified by “guppy classify” as *Pseudomonas aeruginosa* group were renamed to *P. aeruginosa* on the basis of BLAST results for the same reads.

### Phylogenetic grouping of CF specimens

CF specimens were grouped on the basis of the distribution and read mass (the cumulative number of reads contributing to clusters) and were placed onto the CF-unnamed reference tree by “squash” clustering [31] using “guppy squash”, as implemented by *pplacer*. To assess the stability of clades comprised by groups of specimens, we performed 100 bootstrap replicates; resulting trees were summarized using the script *sumtrees.py* as provided in *DendroPy* v3.3.1 [32]. Groups of specimens were defined by considering a combination of branch length, bootstrap support of 70% or greater, the visual cohesiveness of clades, and species composition. The “squash” tree is shown with additional annotation for specimen names and bootstrap support values in **Figure S2**. Seven samples (CF5, CF23, CF37, CF64, CF69, CF71, and CF74) were not assigned to any group because their composition was markedly divergent from other samples in the closest clade. For example, sample CF5, comprised primarily of reads classified as *Acinetobacter* spp., was clearly an outlier from Group II (dominated by *Pseudomonas aeruginosa*) and was therefore not included in that group. Each of the branches corresponding to these unassigned samples (or in the case of CF69/CF37 and CF64/CF71, pairs of samples) was present in 100% of bootstrap replicates, consistent with strong support for their divergence from adjacent clades.

## Results

### Semiconductor sequencing

To efficiently generate sequencing libraries we concatenated sequencing adaptors with PCR primers for amplification of a 16S rRNA sequencing target (variable regions V1–V2), which is sequenced clinically to achieve species-level classification of bacterial pathogens. Forward primers contained a 10-base “barcode” sequence to uniquely label products originating from a specific sample, allowing specimen multiplexing within the same sequencing run.

The length of the target (∼360 bp) and artificial flanking sequences (∼30 bp) exceeded current capabilities of semiconductor sequencing, so it was necessary to develop new protocols to extend the read length. Emulsion PCR, used to prepare template molecules for sequencing by clonally amplifying them on beads, incorporated modified conditions for microdroplet formation and a new PCR enzyme was employed. Sequencing itself was performed using a novel DNA polymerase selected to maximize sequence quality while most efficiently synthesizing longer templates. Flow rates on the sequencing instrument were additionally optimized to improve accuracy and performance for longer template molecules.

**Figure 1:**
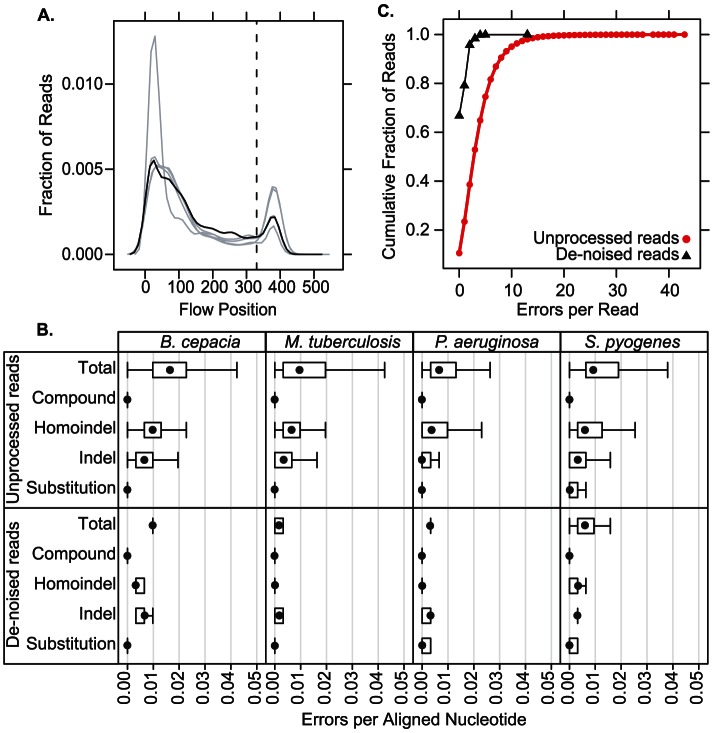
Distribution of read lengths and sequence errors. (**A**) Kernel density plot of read lengths obtained by extended-length ion semiconductor sequencing. Each line represent results from an independent library, black line indicates library containing controls for error rate calculations and sensitivity studies. Vertical line marks the cutoff for full-length sequences. (**B**) Error rates for unprocessed and de-noised sequence reads, stratified by error type and reference organism. (**C**) Cumulative proportion of unprocessed and de-noised sequence reads at defined error counts. For unprocessed reads the fraction of sequences represented at a particular error count reflects the number of reads, and for de-noised sequences it reflects the total number of reads contributing to clusters.

Libraries were pooled from an average of 16 samples each, and were sequenced on a single Ion Torrent PGM using 318 chips. An average of 3,374,183 reads (range = 3,111,252 to 3,681,712) were obtained per chip. Full-length sequence reads comprised ∼18% of all reads (**Figure 1A**). Only full-length reads that could confidently be assigned to a known barcode sequence were considered for downstream analysis. After filtering, we obtained an average of 53,688 reads per sample.

### Sequence error rate, de-noising, and data processing

To assess the per-read error rate we deep sequenced a mixture of DNA from four reference organisms (*Pseudomonas aeruginosa*, *Burkholderia cepacia*, *Streptococcus pyogenes*, and *Mycobacterium tuberculosis*) and compared the reads to corresponding Sanger sequences. The average per-read error rate varied per organism (**Figure 1B**), suggesting sequence-dependent influence on read fidelity. Semiconductor sequencing is prone to insertion and deletion (indel) errors in homopolymer tracts (“homoindels”) [33], and we accordingly found those errors to be most prevalent (averaging 0.8325% per base), exceeding rates of indels in non-redundant sequence (0.435%). Single-base substitution errors were relatively rare (0.07%). Overall errors averaged 1.34% per base per read, similar to published estimates for the Ion Torrent platform [34]–[36].

Molecular classification of species is generally considered to require 98% or greater identity in comparison to a reference sequence [5], [7], while high confidence species-level classification in a clinical setting may require close to 100% identity. Given observed error rates, most individual reads contain too many errors to meet these requirements. Error correction by “de-noising” has been used to make high-throughput sequence data more robust [37], however, existing methods incorporate error models specific to 454 chemistries and are therefore not easily generalizable to semiconductor sequencing [21], [22], [38]. We instead developed a model-free approach to perform error reduction. Briefly, reads were subjected to a modified [21] form of run-length encoding [22], which compressed homopolymer tracts into a representative nucleotide while recording the number of bases in the tract (“run-length”). Encoded sequences were clustered by pairwise identity, and a multiple alignment of each cluster comprised of at least 20 reads was created. Decoded consensus sequences were generated by calculating the most prevalent nucleotide and run-length at each compressed position, then expanding encoded homopolymer tracts accordingly. This approach simultaneously reduces base substitution and indel errors in the resulting consensus sequences. Each consensus sequence is assigned a “read mass” corresponding to the number of reads contained in the clusters that contributed to it.

We identified de-noising parameters (**Figure S1**) that reduced the overall error frequency to an average of 0.633% per base per sequence (**Figure 1B**), and greatly increased the fraction of error-free sequences (**Figure 1C**). De-noising discarded an average of 21.9% of input reads per sample, which were not included in clusters of sufficient size.

### Removal of contaminating sequences

Numerous studies have described exogenous species in 16S rRNA surveys as a consequence of contaminating bacterial DNA in PCR reagents [18], [19]. We performed deep sequencing of non-template and extraction controls to assess this potential. Compared to experimental samples, controls generated limiting quantities of PCR product and were therefore sequenced at 1/100 the concentration of experimental samples. Low numbers of bacterial sequences were obtained from these controls (range 12 to 2476, or 0.02% to 4.2% of the median read count for all clinical specimens) which displayed high similarity to references from *Cupriavidus metallidurans* and *Delftia acidovorans*, organisms with industrial applications [39], [40], and *Pelomonas saccharophila*, *Burkholderia sediminicola/fungorum/bryophila*, *Herminiimonas saxobsidens/glaciei/fonticola*, and *Ralstonia pickettii*, environmental organisms (the latter two also being noted biomedical contaminants [41], [42]), consistent with environmental and/or industrial sources of contamination. To prevent artifactual findings in experimental samples, we therefore removed de-noised consensus reads classified as an organism present in amplification controls. However, contaminating sequences were recovered in only approximately 50% of samples and occurred with low read counts (typically in the tens of reads).

A related issue pertains to the possibility of cross-contamination between specimens. In addition to the low-level of contamination of presumed environmental origin, we amplified sequences from the non-template and extraction control specimens originating from organisms present in high concentration in CF sputa samples (1 to 13 raw reads), consistent with low-level cross contamination from clinical specimens. One specimen (CF38) contained a high concentration of *B. cepacia* as determined by both deep sequencing and by culture. We found a small number of reads in three samples from adjacent PCR wells (one cluster in each of three specimens, ranging from 12–19 reads) that were identified as *B. cepacia*. This finding suggests a low, but detectable, degree of cross-contamination. The cluster size cutoff of 20 reads, found optimal for de-noising, also excluded clusters containing reads attributable to this low level of cross-contamination.

### Recovery of low-prevalence species in polymicrobial specimens and reproducibility

**Figure 2:**
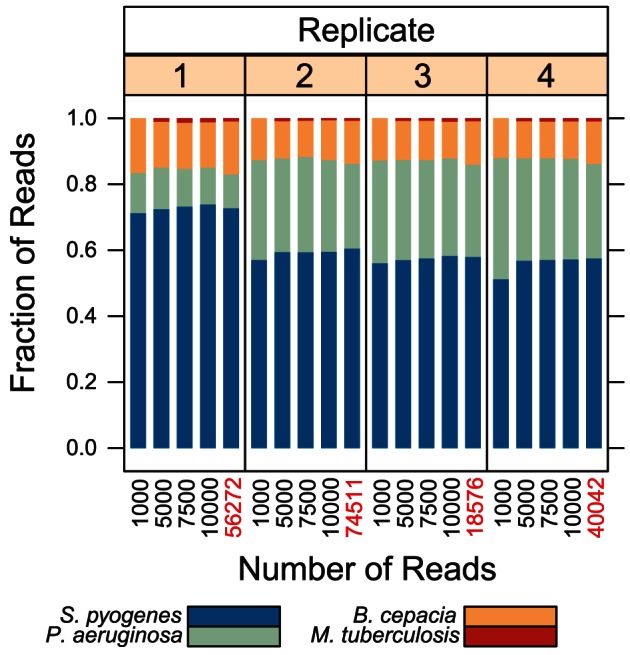
Recovery of low-prevalence species in polymicrobial specimens and reproducibility. The fraction of de-noised sequence reads with highest pairwise alignment scores to the indicated reference sequence among four replicates of sequencing a mixture of reference organisms. Replicates 3 and 4 were generated from 1/10 and 1/100 the template DNA of the other replicates, respectively. The number of de-noised reads (black) or unprocessed reads (red) contributing to each analysis is indicated on the x-axis.

To assess how effectively deep sequencing recovers low-prevalence bacterial species in a complex sample, we sequenced technical replicates of a mixture of purified DNA containing each of the four reference sequences. The estimated relative abundance of each template ranged from 0.25% to 80%. We explored the extent to which detecting minority species is limited by read depth by considering sensitivity given differing numbers of reads randomly subsampled prior to de-noising (**Figure 2**).

The relative representation of organisms was consistent between de-noised and unprocessed reads, across different library preparations of the same control specimen, across the concentrations of initial template DNA used, and among randomly selected subsets of reads of varying sizes. We detected sequences from *Mycobacterium tuberculosis*, which accounted for only 0.25% of 16S rRNA template in the original mixture, in all replicates. The objective of this experiment was primarily to demonstrate sensitivity and technical reproducibility, and we only have estimates of the relative proportion of each of the organisms represented in the mixture. Therefore, we cannot define the precision with which the relative abundance of each organism is reflected by read counts. Bias in the relative amplification efficiency of 16S rRNA from heterogeneous samples is a recognized artifact [43], and metagenomic assays relying on PCR amplification should be considered semi-quantitative. The precise limits of sensitivity in detecting minor species and the extent to which the assay is truly quantitative for a given species is therefore likely dependent on the particular combination of organisms present. However *M. tuberculosis* was consistently detected in simulated down-sampling of experimental data to as few as 5,000 reads, suggesting that depth of sequencing may provide some buffer against failing to detect minority species due to amplification and sampling bias should they exist.

### Characterizing mixed, unculturable clinical specimens

One aim of this study was to assess the performance of deep sequencing relative to existing clinical microbiology techniques. To this end, we deeply sequenced challenging clinical samples and compared our results to those obtained using culture and Sanger sequencing of bulk PCR products (**Table 1**, **Dataset S1**). We focused on specimens that could not be adequately characterized by conventional techniques. To facilitate a direct comparison among methods, we used a BLAST-based classification, requiring at least 99% identity with a reference sequence to assign a classification. Although this conservative approach left a significant fraction of reads in some specimens unclassified at the species level (**Table S3**), it is consistent with criteria used in the clinical laboratory for classification by Sanger sequencing and provides a similar level of confidence in assigned classifications.

**Table 1:** Uncultured clinical specimens and sequencing results.

	Deep Sequencing Results					
Specimen Name/Clinical Sanger Sequencing results	Species name	% of total Reads	Number of Reads	Number of De-noised Clusters	Maximum % Identity	Minimum % Identity
Brain 1/	*Streptococcus constellatus/intermedius*	36.86	11269	5	99.69	99.07
No diagnosis (multiple templates)	No match ≥99%	34.43	10526	29		
	*Porphyromonas endodontalis*	28.55	8728	11	99.68	99.05
	*Streptococcus constellatus*	0.17	52	2	99.08	99.07
Brain 2/	*Staphylococcus epidermidis*	99.01	6874	9	99.68	99.01
No diagnosis (multiple templates)	*Comamonas testosteroni* *	0.69	48	1	100	99.31
	No match ≥99%	0.3	21	1		
Brain 3/	No match ≥99%	44.44	6155	33		
No diagnosis (multiple templates)	*Prevotella oris*	31.62	4379	4	99.37	99.05
	*Porphyromonas endodontalis*	15.6	2161	3	99.68	99.37
	*Streptococcus constellatus/intermedius*	6.28	870	1	99.69	99.08
	*Peptostreptococcus stomatis*	2.06	286	2	99.41	99.12
Brain 4/	No match ≥99%	64.12	11410	24		
No diagnosis (multiple templates)	*Porphyromonas endodontalis*	25.06	4459	12	99.68	99.05
	*Streptococcus constellatus/intermedius*	10.71	1905	2	99.69	99.07
	*Streptococcus constellatus*	0.12	21	1	99.08	99.08
Lymphnode/	*Veillonella parvula/dispar/atypica*	23.6	2742	1	99.7	99.05
*Veillonella* species	No match ≥99%	22.36	2599	17		
	*Fusobacterium periodonticum* *	17.16	1994	2	100	99.32
	*Veillonella dispar/parvula* * */denticariosi*	10.55	1226	3	100	99.07
	*Streptococcus oralis*	5.65	657	2	99.36	99.36
	*Prevotella nanceiensis* *	5.22	607	3	100	99.04
	*Campylobacter concisus*	2.95	343	1	99.03	99
	*Streptococcus parasanguinis*	2.62	304	1	99.68	99.05
	*Peptostreptococcus stomatis*	2.36	274	1	99.71	99.41
	*Streptococcus salivarius/vestibularis/thermophilus*	2	232	1	99.68	99.05
	*Veillonella dispar* * */parvula* *	1.59	185	2	100	99.07
	*Streptococcus pseudopneumoniae/pneumoniae/mitis/oralis*	0.69	80	2	99.68	99.03
	*Rothia mucilaginosa*	0.64	74	1	99.68	99.04
	*Haemophilus parainfluenzae*	0.46	54	1	99.36	99.04
	*Gemella haemolysans*	0.31	36	1	99.69	99.69
	*Streptococcus constellatus* * */intermedius*	0.31	36	1	100	99.38
	*Oribacterium sinus*	0.25	29	1	99.69	99.69
	*Veillonella atypica*	0.24	28	1	99.69	99.69
	*Gemella sanguinis*	0.22	25	1	99.69	99.69
	*Fusobacterium periodonticum/nucleatum*	0.22	25	1	99.66	99.32
	*Capnocytophaga sputigena*	0.22	25	1	99.67	99.02
	*Prevotella melaninogenica*	0.2	23	1	99.68	99.05
	*Streptococcus infantis*	0.2	23	1	99.05	99.05

* 100% identity against reference sequence.

We first sequenced four brain abscess aspirates submitted for conventional molecular characterization by bulk 16S rRNA sequencing. Brain abscesses contain mostly non-viable organisms and therefore frequently fail identification by culture-based techniques [44]. However, they also prove problematic for molecular classification due to the presence of a mixed population of bacterial species translocated from oral and nasopharyngeal cavities [44] contaminated with abundant human cells. Perhaps unsurprisingly, all samples considered here failed culture-based identification and were also un-interpretable by Sanger sequencing. In comparison, deep sequencing confidently identified multiple bacterial species from each specimen with identical or nearly identical BLAST alignments against 16S rRNA reference sequences (**Dataset S1**). Organisms identified were typical of human oral microbiota, including *Streptococcus intermedius*, *Porphyromonas endodontalis*, *Prevotella oris*, and *Peptostreptococcus stomatis*, which have been implicated as relevant organisms in brain abscess formation [44].

We then sequenced a lymph node biopsy for which molecular characterization suggested a *Veillonella* species based on the interpretation of a mixed-appearing, but still interpretable, electropherogram. Deep sequencing confirmed the presence of *Veillonella* species, but identified 16 additional bacterial species not detected by Sanger sequencing, presumably because they were detectable only as minor components of the mixed-appearing background. These findings indicate that even samples that are interpretable by Sanger sequencing may harbor a diverse, and otherwise unrecognized, bacterial population.

### Characterizing cystic fibrosis sputum specimens

Next, we examined sputum samples from cystic fibrosis (CF) patients, whose airways become chronically colonized by a complex mixture of phenotypically variable microbiota [45]. Because such samples are unsuitable for conventional 16S rRNA sequencing, culture remains the standard method for investigating their composition. We deeply sequenced 66 sputum specimens collected from patients seen within the University of Washington's medical system over a 2-month period (March 23 to May 21, 2012). Specimens were submitted either as routine surveillance cultures that are intended to identify specific CF pathogens (for example, *P. aeruginosa* and members of the *B. cepacia* complex) or for identification of causative organisms during acute respiratory exacerbations. Samples were obtained without selection for patient characteristics or clinical indication for culture, and therefore represent a comprehensive sampling of patient samples during this period. These specimens were submitted with an order for “Lower Respiratory Culture for Cystic Fibrosis.” Because these specimens were otherwise de-identified, we cannot confirm the diagnosis of CF, and it is possible that some represent patients with other conditions. In parallel, our CLIA-certified clinical microbiology laboratory performed diagnostic sputum culture according to standard practices, and we performed deep sequencing of DNA purified from the remaining specimen (**Dataset S1**).

We first compared the ability of culture and deep sequencing to identify a targeted panel of CF pathogens of clinical interest, and whose presence in CF patient specimens is routinely evaluated by the clinical laboratory (**Table 2**). Sixty CF sputa were included in this analysis, because culture results were not available for 6 specimens. Public databases of 16S rRNA sequences are well known to contain misclassified, mis-annotated, and otherwise anomalous records [46], so for this analysis we created a carefully curated database of reference sequences limited to organisms of clinical interest in this context and classified de-noised reads using high-stringency BLAST searches as before. Culture and deep sequencing were concordant in most cases, but there were some notable differences. *Stenotrophomonas maltophilia*, *Streptococcus agalactiae*, *Haemophilus influenzae*, and *Pseudomonas aeruginosa* were detected more frequently by deep sequencing than by culture-based methods. Considering results for this set in aggregate, deep sequencing identified specific CF-relevant pathogens with greater frequency than culture (105 from deep sequencing, compared to 94 by culture). Conversely, in 22 cultured organisms (distributed across 17 of the 60 samples) were not reported by deep sequencing, with the most frequent example being *S. aureus*, which was detected by culture alone in 8 separate instances. Six of these missed organisms were recovered in de-noised clusters of less than 20 reads or identifiable using BLAST searches of the raw data (prior to de-noising), suggesting that loss of reads during de-noising at least partially accounts for these failures. Greater sequence read depth would presumably have resulted in detection of the missed organisms in these cases. For the remaining specimens, we found no correlation between failure rate and the relative abundance of the missed organisms based on culture (not shown). We also noted inconsistent mucolysis of unusually thick sputa in several samples, which may have resulted in non-homogenous sample aliquots separately being subjected to culture and DNA extraction.

**Table 2:** CF Pathogens identified by Microbiological Culture and Deep Sequencing.

Organism	Culture Only	Culture and Deep Sequencing	Deep Sequencing Only	Total Cases
*Achromobacter xylosoxidans*		4	1	5
*Burkholderia cepacia* complex		1		1
*Chryseobacterium* species	1			1
*Enterobacter cloacae*		1		1
*Haemophilus influenzae*	1		4	5
*Klebsiella* species	2*			2
*Moraxella catarrhalis*		1		1
*Moraxella nonliquefaciens*		1	1	2
*Mycobacterium abscessus*		1		1
*Mycobacterium avium*			1	1
*Pseudomonas aeruginosa*	2	36	8	46
*Pseudomonas flourescens* group	1			1
*Pseudomonas putida* group	2			2
*Serratia marcescens*	2		1	3
*Staphylococcus aureus*	8	20	4	32
*Stenotrophomonas maltophilia*	3	5	10	18
*Streptococcus agalactiae*		1	3	4
*Streptococcus pneumoniae*		1	†	1
All Organisms	22 (17.3%)	72 (56.7%)	33 (26%)	127 (100%)

* For one case, a single colony of *Klebsiella pneumoniae* was detected by culture.

† 45 patients had consensus sequences with best matches against both *Streptococcus pneumoniae* (pathogen) and *Streptococcus mitis* (normal microbiota). Because such consensus sequences cannot distinguish between these organisms, these instances were not counted.

### Metagenomic analysis of CF sputa

**Figure 3:**
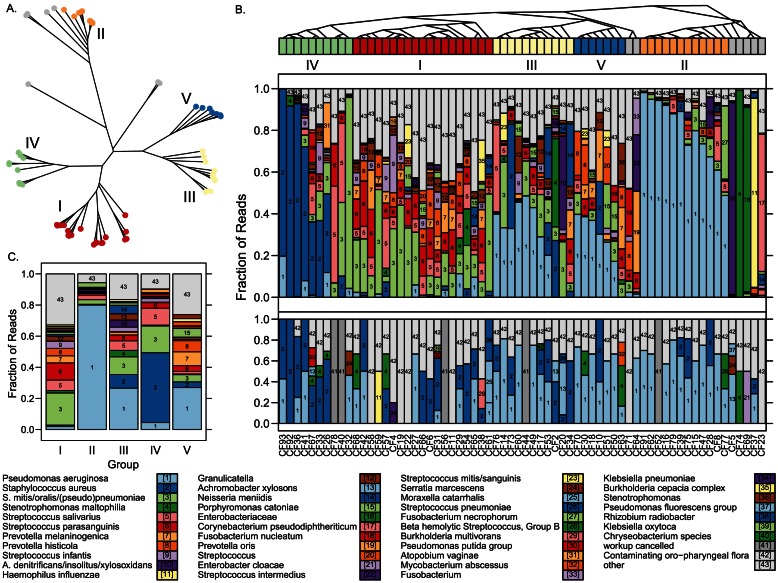
Metagenomic content and phylogenetic clustering of 66 CF sputa samples. Taxonomic names (family, genus, species, or a combination of species where appropriate) appearing with a relative abundance of at least 15% of denoised reads in one or more specimens are indicated in the legend. Any taxonomic name that failed to meet this threshold was assigned the label “Other”. Organisms considered to be components of normal oropharyngeal microbiota by culture were not further speciated according to standard procedures in the clinical laboratory, and were assigned the general label “Contaminating orophoryngeal flora”. Taxonomic labels apply to parts B and C. (**A**) Phylogenetic “squash” clustering of CF bacterial composition. Samples are color-coded according to group (indicated in Roman numerals). Samples colored grey are ungrouped. (**B**) Classification performed by analysis of de-noised deep sequencing reads using *pplacer* (top panel) and culture (bottom panel). The relative number of each species (by read count or colony abundance, respectively) is represented by the height of corresponding bars. Phylogenetic “squash” clustering of specimens from deep sequence data is represented as a cladogram, with specimens colored as in part A. (**C**) Consensus microbiota profile of phylogenetic groups, averaged from all members of the group. Relative abundance of species, as estimated by the fraction of contributory reads, is indicated.

To more fully characterize the bacteria present in CF specimens, and to overcome limitations of a purely identity-based classification approach, we used the *pplacer* [26] software to add de-noised reads to a phylogenetic tree comprised of 16S rRNA reference sequences to support broader classification. As anticipated, when classifying using this larger database, deep sequencing recovered a much larger diversity of organisms than routine methods, including anaerobic and fastidious bacteria expected to be unculturable through standard techniques [47] (**Dataset S1,** **Figure 3C**). A total of 122 species-level classifications were obtained, compared to 18 by culture (sometimes coupled with molecular studies). The organisms most frequently detected among sputum samples from CF patients encompassed both canonical CF pathogens and normal respiratory and oral microbiota, but also included uncommon opportunistic pathogens such as *Corynebacterium pseudodiphtheriticum* [48].

We compared the bacterial communities among CF samples using “squash” clustering [31], which compares specimens based on both the relative abundance and phylogenetic relatedness of organisms (**Figure 3A** **,** **Figure 3B** upper panel , **Figure S2**). Of the 66 sputum samples , 59 could be assigned to one of five major groups, reflecting broad similarities in microbial composition not apparent from culture results (**Figure 3B**, lower panel). Only seven samples could not placed into one of these groups given either their ambiguous placement on the “squash” tree or their metagenomic makeup. Groups were distinguishable from one another by their bacterial composition (**Figure 3C** **, Figure S3, Figure S4**), including a *Pseudomonas*–dominant group (II), a *Staphylococcus* and *Streptococcus*-dominant group (IV), and three distinct, but more heterogeneous groups, composed mostly of *Streptococcus* and *Prevotella* (I), *Streptococcus* and *Pseudomonas* (III), or *Pseudomonas* with *Prevotella* and *Streptococcus* (V).

## Discussion

Next-generation sequencing technologies have gained increasing attention in the field of clinical microbiology [10], [49]. The capability to inexpensively interrogate the full genomes of clinical pathogens holds promise of a transformative effect, offering insight into the molecular biology, molecular epidemiology, and evolution of bacteria that conventional biochemical and morphological classification techniques are incapable of providing. Yet, comprehensive genomic analysis of microbes remains computationally challenging and both time and resource intensive, making the approach prohibitive in the routine clinical environment. Targeted massively parallel sequencing of the 16S rRNA gene is more tractable: limited genotypic information is provided, but allows for phylotypic classification of bacterial species [15]. Deep sequencing of 16S rRNA has already been used numerous times in metagenomic surveys to catalog the taxonomic composition of normal human microbiota [1], [15], and to explore how resident bacterial communities change during various disease states [10]. Regardless, even such targeted genomic sequencing strategies impose practical limitations related to cost, turn-around time, and analytic complexity, precluding their clinical use thus far.

Building upon metagenomic research strategies and existing clinical methods for molecular bacterial characterization, we developed an approach for classifying the species present in clinical samples containing complex bacterial communities using deep sequencing. Semiconductor next-generation sequencing (Ion Torrent) offers rapid chemistries that make it amenable for adaptation as a clinical diagnostic tool, so was selected as the sequencing platform in this study. Subsequent improvements to the workflow with commercial release of Ion Torrent 400 bp sequencing kits have made the assay described theoretically compatible with same day turnaround times (library preparation, 4 hours; automated emulsion PCR, 8 hours; sequencing time, 4 hours; computational analysis time, scalable), potentially allowing for results to be returned faster than can be achieved by culture. In conjunction, multiplexing specimens through DNA barcoding allows significantly reduced per-sample costs [50]: in this study up to 16 samples were run in parallel on a single chip for approximate reagent costs of ∼$60 USD per sample.

We found that sequencing errors for the assay (integrating library construction and sequencing) are largely secondary to artifacts involving indels, a well-known limitation of semiconductor sequencing, and are similar to published error rates for Ion Torrent [34]–[36] (**Figure 1B**). We developed a platform-independent de-noising pipeline that significantly improves overall data quality (**Figure 1B and 1C**) to the point that de-noised sequences from mixed clinical specimens frequently align with 100% identity against bacterial reference sequences (**Dataset S1**), providing the level of accuracy necessary for clinical diagnosis. It should be possible to further decrease errors among de-noised reads by selecting only clusters containing large numbers of reads, but at the expense of decreasing sensitivity secondary to excluding rare sequences.

PCR-mediated deep sequencing library preparation allows highly-purified libraries to be quickly generated from trace quantities of bacterial DNA, in contrast to shotgun sequencing approaches which are less efficient and nonspecifically produce sequence data from the human host [1]. However, PCR results in amplification bias in heterogeneous mixtures due to differences in genomic sequence at primer sites, 16S rRNA copy number, and GC content, such that read counts correlate semi-quantitatively with the relative abundance of bacterial species [43], [51], [52]. However, we observed that it is possible to detect rare bacterial sequences (less than 1%) within complex mixtures of DNA even with a relatively low number of subsampled sequence reads (**Figure 2**). Greater levels of sensitivity are expected if the number of reads dedicated to a specimen is increased.

As an applied proof-of-principle we have explored the composition of challenging clinical specimens, demonstrating key advantages of molecular microbiology diagnosis by next-generation sequencing. Deep sequencing proved most useful in providing actionable information about the microbial composition of brain abscess material, whereas both Sanger sequencing and standard culture failed to provide a result. Similarly, deep sequencing cataloged a number of bacterial species from a biopsy which were not resolvable by Sanger sequencing, and which was clinically reported as infection with a single organism.

In addition to materials where bacteria cannot be effectively cultured or sequenced by the Sanger method, we also explored the utility of deep sequencing using a collection of CF sputa that were simultaneously characterized using standard clinical practice microbiology culture (**Dataset S1**). As expected [47], [53], [54], greater numbers of species-level classifications were obtained by deep sequencing (122 species) than culture (18 species), including fastidious organisms expected to be unrecoverable by routine methods (**Figure 3C**). With respect to detecting specific CF pathogens [55], culture and deep sequencing results agreed in most cases, yet a number of pathogens were detected by deep sequencing in patient specimens deemed to be culture-negative using standard workup (**Table 1**). The limited sensitivity of diagnostic culture when compared to molecular methods, in general, has previously been described for CF pathogens [56], [57]. Even so, 22 of the 127 total pathogens identified were recovered only by culture. *S. aureus* was the organism most frequently missed by deep sequencing, consistent with earlier reports using quantitative real-time PCR [58]. In several cases small numbers of reads were detectable representing the missed pathogen, suggesting that increased read counts would have been sufficient to allow their reliable identification by deep sequencing. Other discrepancies may reflect inefficient DNA extraction from particular organisms, primer bias [43] or properties of the specimens themselves [58], including internal sample heterogeneity. Failures in this study could potentially be addressed by such measures as increasing read depth, optimization of primer design to include additional degenerate sites [59], and controlling pre-analytical variables including sample processing, storage, and DNA extraction [60].

Further optimization will be required before deep sequencing is suitable as a stand-alone diagnostic for CF sputa. Regardless, even currently deep sequencing detected specific CF pathogens from a greater number of patient specimens than culture, indicating utility as an adjunct identification technique. Moreover, members of the *Streptococcus milleri* group (*S*. *anginosus*, *constellatus and intermedius*), CF pathogens that are not resolved by routine clinical culture [47], were confidently classified by deep sequencing in 25 patient samples (**Dataset S1**). Thus, the true number of CF pathogens diagnosable by deep sequencing is greater than reported with respect to the limited panel of organisms surveyed by culture.

It may prove more informative to evaluate the overall microbial population in a patient's airway rather than to screen for specific pathogens [45], [61]. We therefore compared the microbiota of 66 CF sputa, demonstrating for the first time the feasibility of rapid metagenomic classification as a clinical diagnostic. We found that CF samples in this study can largely be divided into five major groups based only on similarities in their microbial composition (**Figure 3** **, Figure S3, Figure S4**), which are not apparent based on conventional culture results. This finding suggests that a diverse CF patient population can be binned into a limited number of categories given the makeup of their respiratory microbiota. Two of the groups (II and IV) have relatively low diversity and are dominated by combinations of *Staphylococcus*, *Streptococcus*, and *Pseudomonas*; all well-described colonizers of the airway of CF patients. Groups I, III, and V are more diverse. Groups I and V each contain a substantial fraction of obligate anaerobes including *Prevotella*, *Veillonella*, and *Porphyromonas* species. Anaerobic organisms have been noted in CF sputa in a number of studies [62], [63], although their clinical significance is uncertain. In contrast, group III has a smaller representation of anaerobes. Whether the presence or absence of particular metagenomic profiles will correspond meaningfully with clinical correlates remains to be seen, but the finding opens exciting possibilities for a future paradigm shift in clinical microbiology from the identification of single organisms to diagnoses based on the overall population content of a sample [64]. Additional studies will be required to reproduce and provide statistical support for these groups.

There are several additional considerations to the use of 16S rRNA deep sequencing in the clinical laboratory. First, although de-noising strategies have proven valuable, their use prevents discrimination among closely related strains. Because de-noising functions by clustering similar reads that are assumed to derive from the same template molecule, sufficiently similar sequences may be integrated into a single consensus. Therefore, although our approach can accurately and sensitively “rule in” bacteria whose sequences closely match those in a database of known 16S rRNA genes, it currently does not allow certain bacterial species to be “ruled out” from clinical specimens in cases where a closely related species is also detected. We expect that future improvements in PCR enzyme cocktails, sequencing chemistries, and primary base-calling algorithms will reduce rates of raw sequencing error on this platform, decreasing reliance on de-noising algorithms and improving the resolution of the assay. More sophisticated de-noising algorithms incorporating error models specific to semiconductor sequencing may also prove beneficial [21], [22], [38]. Secondly, our method relies on classifying experimental sequences against a defined set of 16S rRNA references, which greatly limits the potential for spurious classification due to sequencing errors [65], [66] but also makes the discovery of previously un-described organisms more challenging. Further, although the assay is able to detect low prevalence bacteria in multi-component specimens with previously unachievable sensitivity, this property also presents challenges. In many cases the presence of particular minor bacterial species might have unclear diagnostic implications, especially if the organism is a pathogen at the limits of detection, and additional studies will be needed to explore the significance of such findings. From a practical standpoint, extreme sensitivity also makes the approach susceptible to contaminating DNA and special care must be employed to avoid this, along with inclusion of appropriate extraction and non-template controls. We should note that the pilot experiments described in this study were performed in the absence of fully realized environmental controls that we expect would be in place for a clinically-validated assay to minimize the risk of specimen cross-contamination. Lastly, in some situations only genus or multiple species-level classifications can be assigned due to insufficient discriminatory information the 16S rRNA gene V1–V2 regions. As read lengths offered by semiconductor sequencing increase, it may be possible to interrogate more of the 16S rRNA gene in the future.

Despite these caveats, deep sequencing demonstrates the potential for immediate utility in several clinical applications exemplified by this study, namely, characterizing mixed infections from specimens containing non-viable or unculturable organisms, such as brain abscesses or fixed tissues, and detecting specific bacterial pathogens from complex specimens when a defined list of species are of interest, such as CF sputa [58]. Further work will be required to more fully catalog the range of bacteria detectable in various disease states and to correlate the presence of particular agents with patient outcomes before deep sequencing can fully inform patient care as a general molecular diagnostic, independent of the clinical indication.

## Supporting Information

Dataset S1 **Classification results for clinical specimens.**
(XLSX)

File S1 **De-noised sequences.**
(TGZ)

File S2 **CF-named reference package.**
(BZ2)

File S3 **CF-unnamed reference package.**
(BZ2)

Figure S1 **Overall error rates for different de-noising parameters.**
(PDF)

Figure S2 **Squash clustering of CF sputa microbiota. Bootstrap support values are indicated along corresponding nodes.**
(PDF)

Figure S3 **Genus-level classification performed by analysis of de-noised deep sequencing reads using** ***pplacer*** **.** The relative number of each species (by read count) is represented by the height of corresponding bars. Phylogenetic “squash” clustering of specimens from deep sequence data is represented as a cladogram, with specimens colored as in Figure 3.
(PDF)

Figure S4 **Consensus microbiota profile of phylogenetic groups at the genus-level, averaged from all members of the group.**
(PDF)

Table S1 **Primer Sequences.**
(XLSX)

Table S2 **Composition of sequencing libraries.**
(XLSX)

Table S3 **Putative genus-level classification of consensus sequences from brain abscesses and lymph node biopsy that were unassigned at the species-level.**
(XLSX)
