# Supplementary figures and images for: Rapid 16S rRNA Next-Generation Sequencing of Polymicrobial Clinical Samples for Diagnosis of Complex Bacterial Infections
Source: PLoS One. 2013 May 29;8(5):e65226. doi: 10.1371/journal.pone.0065226 (PMC3666980; doi:10.1371/journal.pone.0065226)

Errors Per Base

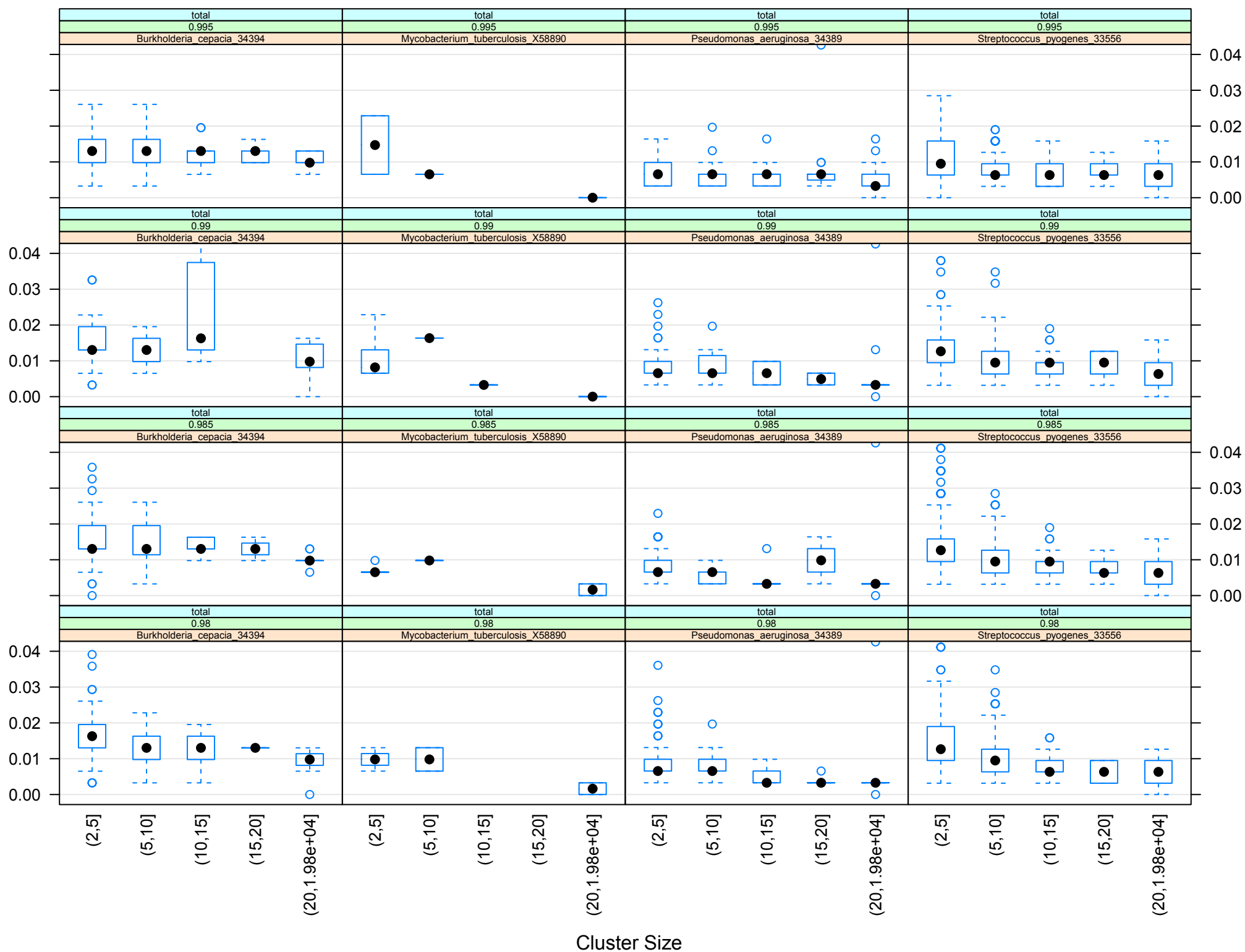

Supplement: Figure S1 — Overall error rates for different de-noising parameters. (PDF) [file pone.0065226.s005.pdf]

11

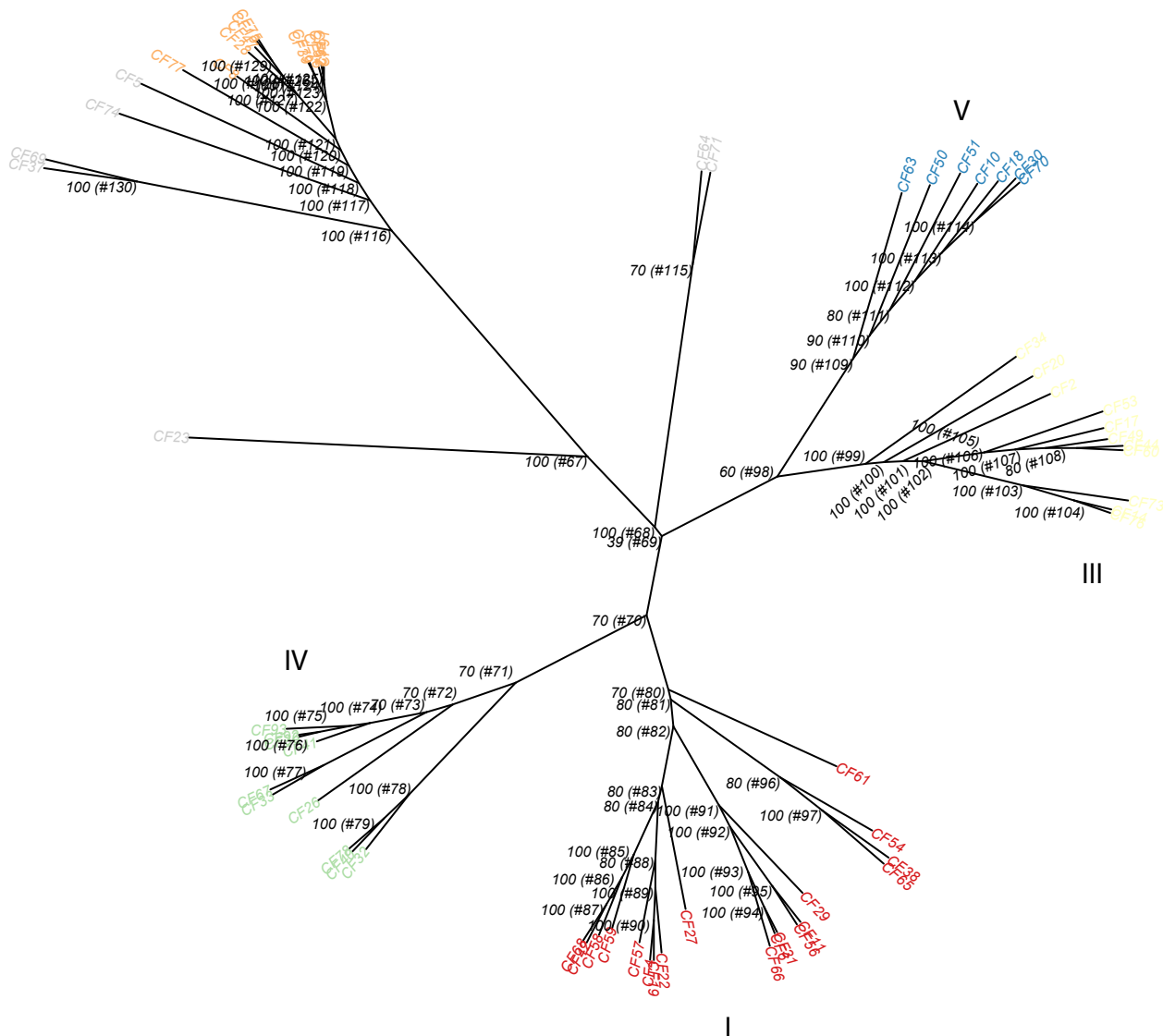

Supplement: Figure S2 — Squash clustering of CF sputa microbiota. Bootstrap support values are indicated along corresponding nodes. (PDF) [file pone.0065226.s006.pdf]

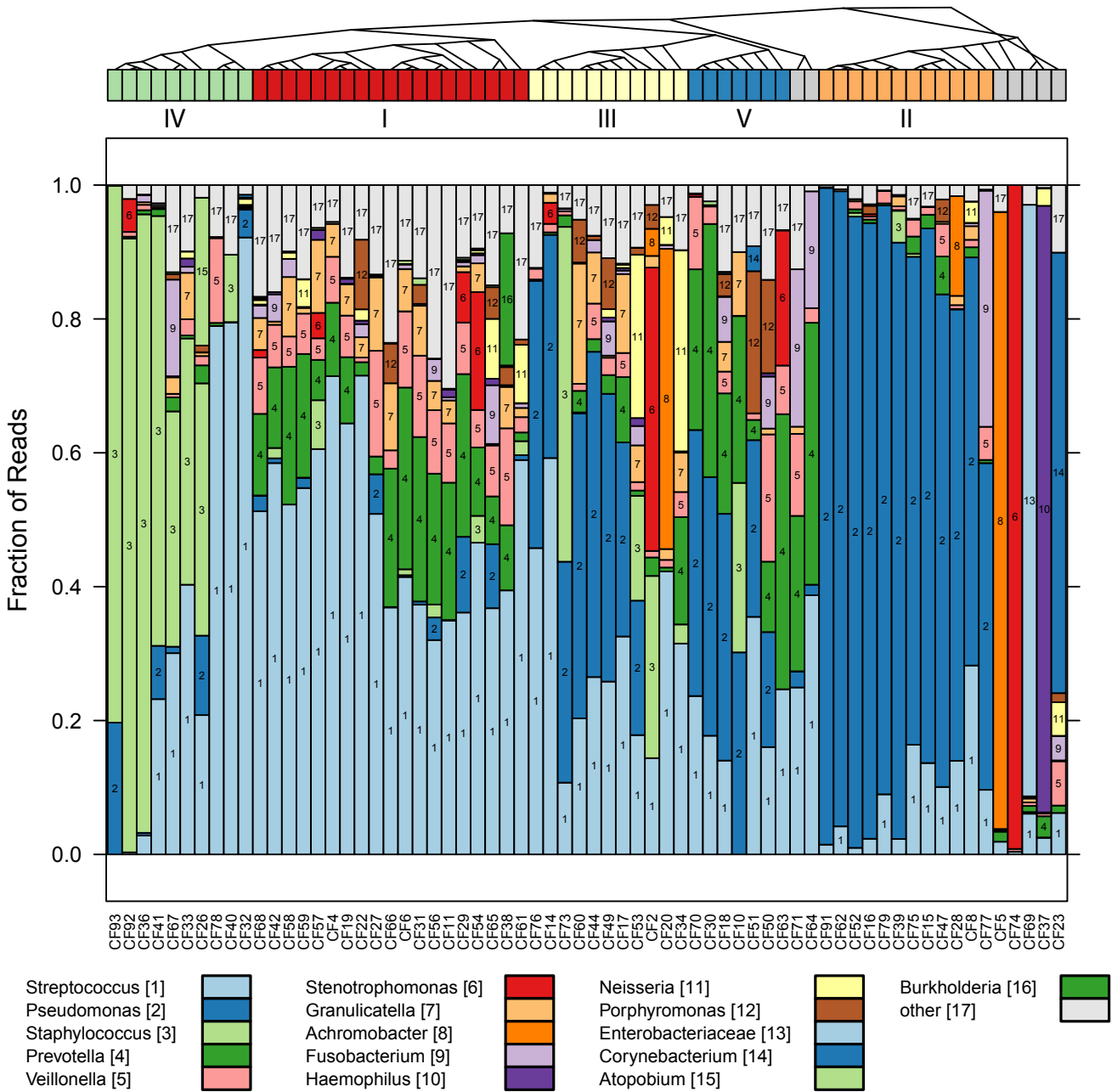

Supplement: Figure S3 — Genus-level classification performed by analysis of de-noised deep sequencing reads using pplacer . The relative number of each species (by read count) is represented by the height of corresponding bars. Phylogenetic “squash” clustering of specimens from deep sequence data is represented as a cladogram, with specimens colored as in Figure 3. (PDF) [file pone.0065226.s007.pdf]
