# Supplementary material for: Rapid 16S rRNA Next-Generation Sequencing of Polymicrobial Clinical Samples for Diagnosis of Complex Bacterial Infections
Source: PLoS One. 2013 May 29;8(5):e65226. doi: 10.1371/journal.pone.0065226 (PMC3666980; doi:10.1371/journal.pone.0065226)
Supplement: Figure S4 — Consensus microbiota profile of phylogenetic groups at the genus-level, averaged from all members of the group. (PDF) [file pone.0065226.s008.pdf]

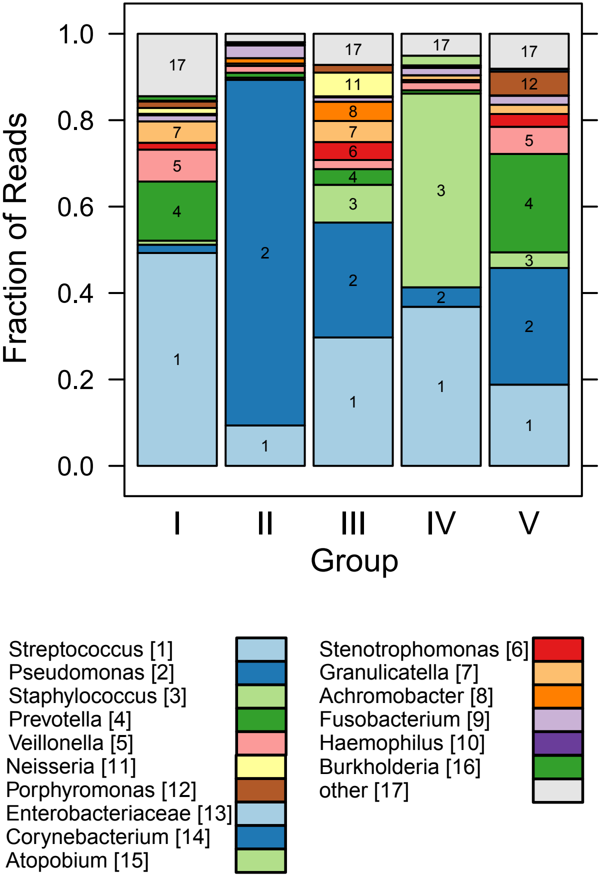
1.0
0.8
0.6
0.4
0.2
0.0
Fraction of Reads
I
II
III
IV
V
Group
17
7
5
4
1
2
1
11
8
6
3
3
12
Streptococcus [1]
Pseudomonas [2]
Staphylococcus [3]
Prevotella [4]
Veillonella [5]
Neisseria [11]
Porphyromonas [12]
Enterobacteriaceae [13]
Corynebacterium [14]
Atopobium [15]
Stenotrophomonas [6]
Granulicatella [7]
Achromobacter [8]
Fusobacterium [9]
Haemophilus [10]
Burkholderia [16]
other [17]
